# The Exploration of Peptide Biomarkers in Malignant Pleural Effusion of Lung Cancer Using Matrix-Assisted Laser Desorption/Ionization Time-of-Flight Mass Spectrometry

**DOI:** 10.1155/2017/3160426

**Published:** 2017-03-13

**Authors:** Jing Xu, Bin Xu, Chuanhao Tang, Xiaoyan Li, Haifeng Qin, Weixia Wang, Hong Wang, Zhongyuan Wang, Liangliang Li, Zhihua Li, Hongjun Gao, Kun He, Xiaoqing Liu

**Affiliations:** ^1^Department of Pulmonary Oncology, 307 Hospital, PLA, Beijing, China; ^2^National Center of Biomedical Analysis, Beijing, China; ^3^Department of Tuberculosis, 309 Hospital, PLA, Beijing, China; ^4^Department of Oncology, General Hospital of the PLA Rocket Force, Beijing, China

## Abstract

*Background*. Diagnoses of malignant pleural effusion (MPE) are a crucial problem in clinics. In our study, we compared the peptide profiles of MPE and tuberculosis pleural effusion (TPE) to investigate the value of matrix-assisted laser desorption/ionization time-of-flight mass spectrometry (MALDI-TOF-MS) in diagnosis of MPE.* Material and Methods*. The 46 MPE and 32 TPE were randomly assigned to training set and validation set. Peptides were isolated by weak cation exchange magnetic beads and peaks in the *m*/*z* range of 800–10000 Da were analyzed. Comparing the peptide profile between 30 MPE and 22 TPE samples in training set by ClinProTools software, we screened the specific biomarkers and established a MALDI-TOF-MS classification of MPE. Finally, the other 16 MPE and 10 TPE were included to verify the model. We additionally determined carcinoembryonic antigen (CEA) in MPE and TPE samples using electrochemiluminescent immunoassay method.* Results*. Five peptide peaks (917.37 Da, 4469.39 Da, 1466.5 Da, 4585.21 Da, and 3216.87 Da) were selected to separate MPE and TPE by MALDI-TOF-MS. The sensitivity, specificity, and accuracy of the classification were 93.75%, 100%, and 96.15%, respectively, after blinded test. The sensitivity of CEA was significantly lower than MALDI-TOF-MS classification (*P* = 0.035).* Conclusions*. The results indicate MALDI-TOF-MS is a potential method for diagnosing MPE.

## 1. Introduction

Lung cancer is an aggressive malignancy, often accompanied by pleural metastasis [1]. It is reported that lung cancer is the most common pathogen of malignant pleural effusion (MPE) [2, 3], and more than 50% of the patients developed pleural effusion during their disease course [1, 4]. The emergence of MPE indicates the patients lose the opportunity of operation and have poor prognosis [5].

Cytological detection is still the main method for diagnosis of MPE, but with a low positive rate (40%–70%) [5–7]. Moreover, a series of tumor biomarkers such as CEA, CY21-1, and CA125 [8–10] also help to diagnose MPE in clinical practice, but their sensitivity and specificity are not high enough to meet the clinical demand. The lack of effective diagnostic methods can result in underestimation of the disease's stage, inadequate treatment, and affecting the prognosis of patients. So finding an alternative method for diagnosis of MPE is of great importance now.

Exudative pleural effusion is a kind of protein-rich fluid, the majority of which are high abundant proteins from plasma, others such as proteins secreted by tumor cells, proteins released by dead cells, and membrane proteins [5, 11, 12]. Most of these proteins are unfamiliar to us and may be associated with specific tissue or disease. Therefore, it is a promising way to explore potential biomarkers related to malignancy in MPE based on proteomics.

Nowadays, the proteomic technology is being widely used in biomarkers research. Screening new potential protein biomarkers in body fluid plays an important role in disease diagnosis and efficacy prediction. In our study, we use a modern technology, matrix-assisted laser desorption/ionization time-of-flight mass spectrometry (MALDI-TOF-MS) to explore protein/peptide biomarkers. What distinguishes this method from other traditional proteomic technologies is that it is more stable, convenient, sensitive, and simple to operation [13]. Furthermore, low-abundant peptides extracted by magnetic bead-based immobilized metal ion coupling with MALDI-TOF-MS are more likely to be associated with disease.

The purpose of our study is to explore potential protein/peptide biomarkers and establish a new diagnostic classification of MPE by comparing the different peptide profiles of MPE of lung cancer and TPE based on MALDI-TOF-MS in combination with weak cation exchange magnetic beads (MB-WCX).

## 2. Material and Methods

### 2.1. Patients and Samples

The lung cancer patients were from the Department of Lung Cancer of Affiliated Hospital of Academy of Military Medical Science between October 2013 and October 2014; all of the patients were diagnosed with adenocarcinoma by pathology/cytology and all of the patients developed PE. The PE sample was required to meet the following criteria: (1) All of PE samples were exudative pleural effusion diagnosed by Light's criteria. (2) Patients should have none of the following complications: obstructive pneumonia, atelectasis, and pulmonary embolism. (3) Patients with active infection, second primary tumors, and other diseases such as heart, liver, kidney dysfunction, and connective tissue diseases were excluded. (4) All of samples had been tested for cytological smear. (5) Patients did not receive any intrapleural therapy except thoracentesis.

A total of 66 PE samples of lung cancer patients were collected according to the above criteria. Smears from 46 PE samples (69.70%) showed adenocarcinoma cells, while we did not find any malignant cells in the other 20 PE samples (30.3%).

The patients with tuberculous pleurisy were from The 309 Hospital of PLA between October 2013 and June 2014. The patients should meet the following criteria: (1) There were typical signs and symptoms and radiologic evidences in support of the diagnosis of tuberculous exudative pleurisy. (2) The result of purified protein derivative (PPD) skin test was strongly positive. (3) The antituberculosis treatment was effective. (4) The patients with other nontuberculosis disease were excluded. (5) Pleural biopsy revealed tuberculous granuloma or the result of acid fast staining was positive. (6) All of PE samples were exudative pleural effusion diagnosed by Light's criteria. (7) The patients with any tumor disease and receiving any intrapleural administration were excluded.

A total of 32 TPE samples were collected, and each TPE sample was also examined by cytological smear method to exclude the tumor disease. The clinical characteristics of the patients in the study were shown in Table 1.

All of the PE samples were obtained by thoracentesis after ultrasound localization and every patient wrote informed consent prior to collection of samples. Liquid supernatant of PE samples was separated by centrifugation at 4000 rpm for 10 minutes at 4°C after being set aside for 2 h, then separated into aliquots (100 *μ*L each) immediately, and frozen at −80°C until further analysis.

### 2.2. MALDI-TOF Mass Spectrometry

#### 2.2.1. Grouping

The training set included 30 MPE samples and 22 TPE samples randomly selected from 46 cytological positive MPE samples and 32 TPE samples, respectively, at the ratio of 2 : 1, while the remaining 16 MPE samples and 10 TPE samples consisted of the validation set to test the results. Besides, the other 20 PE samples of lung cancer patients which were negative in cytological examination were also analyzed by MALDI-TOF-MS.

#### 2.2.2. Peptides Isolation

Peptides were purified by weak cation exchange magnetic beads (MB-WCX, National Center of Biomedical Analysis, China) after the liquid supernatant of PE samples was thawed gradually. All of the process was according to the standards procedure of manufacturer. The first step was binding the peptides to magnetic beads: put 5 *μ*L magnetic beads in 50 *μ*L binding solution (National Center of Biomedical Analysis, China) for washing three times; then 5 *μ*L PE sample and 20 *μ*L binding solution were added to the washed magnetic beads; the sample was incubated at room temperature for 10 minutes. The above process was exchanged on the magnetic bead separation device three times and the supernatant was abandoned. The second step was washing the nonproteins and high abundant proteins off the beads: use 100 *μ*L washing solution (National Center of Biomedical Analysis, China) to wash the beads three times on magnetic bead separation device and discard the supernatant. The third step was eluting the bound peptides: 20 *μ*L eluting solution (National Center of Biomedical Analysis, China) was added to the beads and incubated at room temperature for 20 minutes; the sample was exchanged on the magnetic bead separation device three times for the obtainment of peptides elution.

#### 2.2.3. MALDI-TOF-MS Analysis

Saturated *α*-cyano-4-hydroxy-cinnamic acid (*α*-HCCA, Bruker Daltonics, Germany) prepared in 0.1% trifluoroacetic acid (TFA, Sigma-Aldrich, USA) and 50% acetonitrile (ACN, Sigma-Aldrich, USA) composed the matrix solution. The mixture of 0.5 *μ*L matrix solution and 0.5 *μ*L peptides elution was spotted on AnchorChip target plate (Bruker Daltonics, Germany) and allowed to dry on the plate at room temperature. The intensity of peaks was corrected by external calibration: the mixture of 0.5 *μ*L matrix solution and 0.5 *μ*L Peptide Calibration Standard Product (including angiotensin I (*m*/*z* 1,297.49), angiotensin II (*m*/*z* 1,047.19), substance P (*m*/*z* 1,348.64), ACTH clip 18–39 (*m*/*z* 2,466.48), ACTH clip 1–17 (*m*/*z* 2,094.43), bombesin (*m*/*z* 1,620.86), and somatostatin (*m*/*z* 3,149.57), Bruker Daltonics, Germany) was also spotted on AnchorChip target plate for calibration.

Each spot was scanned by the laser of Ultraflex III matrix-assisted laser desorption/ionization time-of-flight mass spectrometer (MALDI-TOF-MS) (Bruker Daltonics, Germany) with a frequency of 200 Hz on linear positive ion mode. The ion source voltages 1, 2 and lens voltage of the instrument were 25 kV, 23.50 kV, and 6.5 kV, respectively. Laser intensity was set to 43% of the maximum value and *m*/*z* range from 800 Da to 10000 Da was monitored by FlexControl acquisition software v3.4 (Bruker Daltonics, Germany). 500 laser shots were pulsed on six different positions at each sample spot randomly and the pulsed ion extraction time was 100 ns (the total shots were 3000).

#### 2.2.4. Biostatistics

All of the spectral data were processed by ClinProTools software v2.1 (Bruker Daltonics, Germany). First, the spectral data were normalized to their total ion count after baseline subtraction. Then, recalibrate the data to reduce the mass shifts. The peak areas of total average spectrum and individual spectrum were finally calculated, and the peaks were detected on the total average spectrum when signal-to-noise ratio was 5. The majority of *m*/*z* of resolved peptides were mainly within the range of 800–10000 Da. As the *m*/*z* was higher than 10000 Da, we cannot detect high signal peaks, while the *m*/*z* lower than 800 Da were also excluded because most of them were signal noises of other molecules.

The peptide spectral peaks of MPE and TPE in training set were compared and different peaks whose areas under the curve were statistically significant between MPE and TPE were identified. ClinProTools software v2.1 supported three kinds of statistical algorithms: mathematical models genetic algorithm (GA), Supervised Neural Network (SNN), and quick classifier algorithm (QC). Each of the three algorithms selected a particular combination of peptide peaks to generate the classification model. Then the performance of an algorithm was described by recognition capability, and the performance of the model was evaluated by a cross-validation process repeatedly within the software. We chose the optimal model with high performance according to the above two values.

In order to predict the capability of the calculated model, a blind external validation was performed. ClinProTools software requires a new set of spectra for validation, so another new set of MPE and TPE samples were prepared and loaded in the same way as the samples processed in training set and then were classified against the model. Corresponding spectrum of each sample in validation set was made to challenge the classification model. The PE samples classified as malignant pleural effusion by the MALDI-TOF-MS classification were then labeled “malignant,” while those classified as tuberculosis pleural effusion by the model were labeled “benign.” The samples were labeled “unclassifiable” if their spectra were null and unclassifiable.

### 2.3. Detection of CEA in PE Samples

We examined CEA in 31 MPE samples and 32 TPE samples using electrochemiluminescent immunoassay method in Clinical Laboratory of Affiliated Hospital of Academy of Military Medical Science. The recommended cut-off value is 4.3 ng/mL (CEA > 4.3 ng/mL is positive, and CEA < 4.3 ng/mL is negative).

### 2.4. Statistical Analysis

The comparison of clinical characteristics and the positive rate between different groups was done using *χ*^2^ or Fisher's exact test. Statistical analyses were performed using IBM SPSS statistics version 19 software (SPSS Inc., USA). *P* < 0.05 was considered statistically significant difference. The comparison of the area under the peptide peaks between different groups was done using *t*-test with ClinProTools software (version 2.1).

## 3. Results

### 3.1. The Clinical Characteristics of All Patients between MPE and TPE Group

In our study, 98 PE samples met the enrollment criteria. Among that, 66 PE samples of lung cancer patients were diagnosed as malignant pleural effusion by clinical judgment initially, and 32 PE samples of tuberculous pleurisy patients were diagnosed as tuberculosis pleural effusion by pleural biopsy. All of the 66 samples of lung cancer patients were examined by cytological smear, and 46 (69.70%) samples were discovered malignant cells. 32 PE samples of tuberculous pleurisy patients were also examined by cytological smear to exclude neoplastic disease.

The general clinical characteristics of all patients were shown in Table 1. The median age of patients with MPE was 61 years old (36–82 years old), and media age of patients with TPE was 29 years old (15–96 years old). There was more bloody appearance in MPE samples (42/66) than TPE samples (2/32, *P* < 0.0001). The gender and smoking status were balanced between TPE and MPE patients (Table 1).

### 3.2. The Difference Peptide Profiles between MPE and TPE in the Training Set

**Figure 1:**
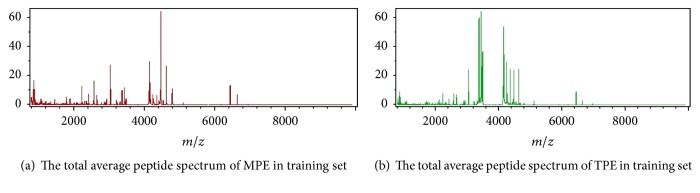
The total average peptide spectra of the training set displayed by ClinProTools software.

**Figure 2:**
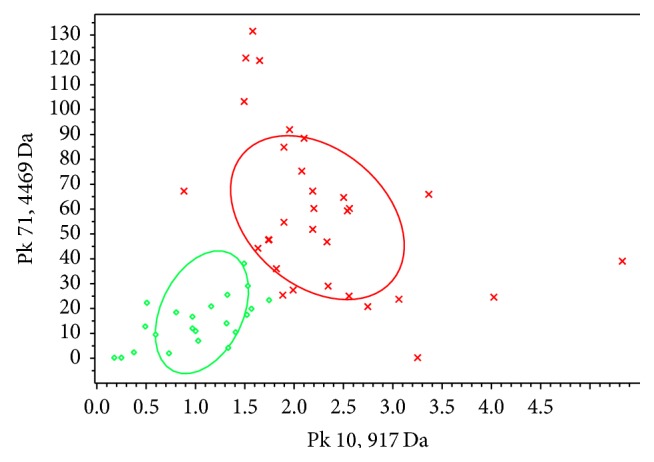
2D peak distribution view of peptides with *m*/*z* 917 Da (*x*-axis) and 4469 Da (*y*-axis) between malignant pleural effusion (red cross) and tuberculosis pleural effusion (green circle) in training set by ClinProTools software V2.1. 917 Da and 4469 Da are the most significant different peaks in MPE and TPE. The coordinate scale stands for peptide abundance ratio and the two circled areas are the standard deviation of the class average of peak area.

Training set included 30 MPE and 22 TPE. The total average peptide spectra of MPE and TPE analyzed by ClinProTools software were shown in Figure 1. Most of the spectral peaks were similar in the two classes, while there were also subtle differences which can be potential biomarker candidates. A further comparative analysis acquired 94 different peptide peaks in the 800~10000 Da range between MPE and TPE in training set. A total of 28 peptide peaks were of statistics significance (*P* < 0.05). Among them, 15 peaks presented a higher peak area in MPE and the other 13 peaks presented a lower peak area in MPE (Table 2). The two peaks (*m*/*z* 917 Da and 4469 Da, *P* < 0.001) which were of the most significant difference in MPE and TPE were designated as the *x*- and *y*-axes, respectively, to draw a 2D peak cluster distribution map (Figure 2). 917 Da and 4469 Da were considered as two of the most important peaks in the classification model.

### 3.3. Establishing the MALDI-TOF-MS Classification Model

The three kinds of algorithm embedded in the ClinProTools software—SNN algorithm, GA algorithm, and QC algorithm—were applied to establish the classification model, respectively, using the peptide peaks of training set. The SNN algorithm which showed the best performance on distinguishing MPE samples from TPE samples was the optimal algorithm in that the recognition rate was 98.44% and the cross-validation rate was 81.06% (Table 3).

**Figure 3:**
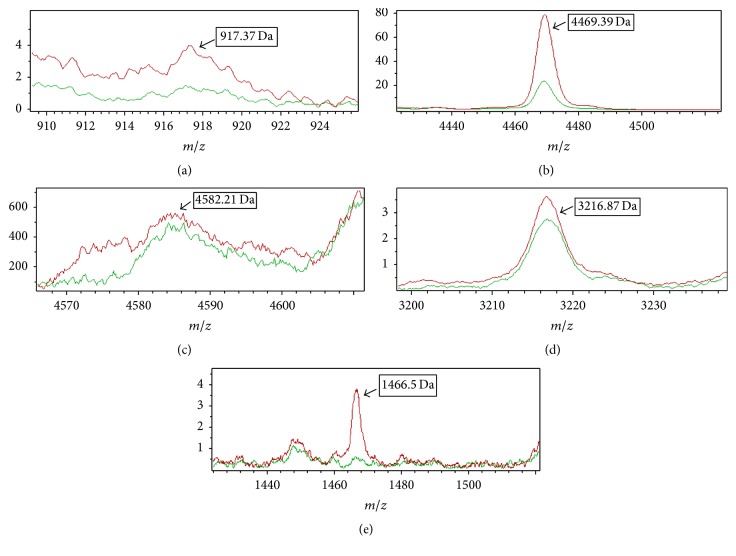
The average intensity of five peptides composing the classifier with malignant and tuberculosis pleural effusion showed by ClinProTools software (the red line represents malignant pleural effusion; the green line represents tuberculosis pleural effusion).

The classification model established by the SNN algorithm consisted of five peptide peaks: 917.37 Da, 4469.39 Da, 1466.5 Da, 4585.21 Da, and 3216.87 Da (Figure 3, Table 4).

All of the five peptide peaks were upregulated in malignant pleural effusion. It can be defined as “malignant” when a PE sample met the following conditions: the peptide peak area of 917.37 Da was in the range of 22.25 ± 8.730 Da, the area of 4469.39 Da was in the range of 562.6 ± 326.2 Da, the area of 1466.5 Da was in the range of 23.23 ± 16.64 Da, the area of 4585.21 Da was in the range of 21.55 ± 10.81 Da, and the area of 3216.87 Da was in the range of 28.27 ± 13.60 Da.

It can be defined as “benign” when the peptide peak area of a PE sample was in the range of 10.56 ± 4.680 Da of the 917.37 Da, 184.1 ± 247.9 Da of 4469.39 Da, 8.200 ± 4.920 Da of 1466.5 Da, 14.84 ± 7.360 Da of 4585.21 Da, and 25.21 ± 13.85 Da of 3216.87 Da.

### 3.4. Blind Test of the MALDI-TOF-MS Classification Model in Validation Set

Our classification model was validated by another new set of 16 MPE samples and 10 TPE samples. As a result, all of the 10 TPE samples confirmed by pleural biopsy were labeled as “benign,” while, among the 16 MPE samples confirmed by cytological smear, 15 samples were labeled as “malignant” and a sample which cannot be classified was labeled “unclassifiable.” The sensitivity and specificity of our classification were 93.75% (15/16) and 100.00% (10/10); the accuracy of the classification was 96.15% (25/26) (Table 5).

In addition, we analyzed 20 PE samples of lung cancer patients which were cytologically negative but were diagnosed as MPE by clinical judgment for the high false negative rate of cytological smear. Among the 20 PE samples, two samples with null spectra were labeled “unclassifiable.” And in the 18 remaining samples, 16 (88.88%) samples were also labeled as “malignant” by MALDI-TOF-MS classification model.

### 3.5. The Comparison between MALDI-TOF-MS Classification Model and Cytological Smear

A total of 66 PE samples of lung cancer patient were measured with cytological smear. The malignant cells were found in 46 cases (69.70%) and the other 20 PE samples were cytologically negative. As mentioned, we totally analyzed 36 PE samples of lung cancer patients by MALDI-TOF-MS. Among the 33 samples that yielded valid spectra, 31 PE samples (93.94%) were labeled “malignant” and only 2 samples were labeled “benign.” The comparison of these two methods was shown in Table 6; the detection rate of MALDI-TOF-MS classification model was higher than traditional cytological smear method (*P* = 0.006).

### 3.6. The Comparison between MALDI-TOF-MS Classification Model and CEA Detection

To ensure the accuracy of the result, we only chose the MPE samples which were diagnosed by cytological smear. Among the 46 MEP samples diagnosed by cytological smear, 31 MPE samples were measured CEA: 21 MPE samples (67.74%) were positive, and 10 MPE samples (32.26%) were negative (the cut-off value is 4.3 ng/mL). Among the 32 TEP samples, 9 TPE samples (28.13%) were positive, and 23 cases (71.87%) were negative. The sensitivity and specificity of CEA test in our study were 67.74% and 71.87%.

The results of CEA detection and MALDI-TOF-MS classification model were shown in Tables 7 and 8. The sensitivity of CEA was significantly lower than MALDI-TOF-MS classification (*P* = 0.035), but the specificity was of no statistical difference (*P* = 0.147).

## 4. Discussion

In China, the metastasis of malignant tumors is the second common cause of exudative pleural effusion only after tuberculous pleurisy. The cytological examination is still the gold standard for the diagnosis of malignant pleural effusion currently. However, it is reported that its false negative rate is about 31.5%, which can not meet the demands of the clinical work [5, 14–16].

Exudative PE has abundant protein content and many of the proteins are associated with specific disease which are released by specific cells and pulmonary tissues. Therefore, using the comparative proteomic technique to analyze the different proteomic profiles and then find the pathogenesis of disease is of extensive value.

Tissue and body fluid samples are widely used to screen tumor biomarkers in clinical application now. The tissue samples, however, are often inadequate for screening biomarkers and dynamic analysis because of their small quantity, low tumor content, or being not very readily available [17]. Compared with tissue samples, liquid samples become increasingly popular for its easy accessibility and dynamic monitoring.

Now the blood samples (plasma or serum) are widely applied to screen tumor biomarkers based on MALDI-TOF-MS and have made some achievements. In our previous study, three peptides (7,478.59 Da, 2,271.44 Da, and 4,468.38 Da) had been screened out to build a diagnosis model of NSCLC through MALDI-TOF-MS analysis by the comparison between non-small cell lung cancer (NSCLC) patients and healthy people on their serum protein/peptide profile [18]. The model was highly sensitive (100%) and specific (96.9%). According to our recent study, MALDI-TOF-MS can also differentiate the small variations between different serum peptide profiles of NSCLC patients with different EGFR Gene Mutation Status [19]. These two previous studies demonstrated the feasibility of this method and offered some technical and practical experiences for further research. Although blood samples contain the substances of the primary lesions and systemic metastases lesions, it is difficult to screen out the tumor biomarkers due to the low concentration. But the pleural effusion sample, which is closer to the affected pulmonary tissue and hence more specific for pulmonary diseases than other body fluids, contains plasma proteins and proteins associated with lung cancer [20]. To our knowledge, there are few studies that analyzed the discriminating peptide profiles of pleural effusion samples based on MALDI-TOF-MS. Our study compared the differential peptide profiles of malignant (only MPE from lung adenocarcinoma patients) and benign inflammatory pleural effusions (only tuberculosis pleural effusion as control group) to screen a panel of specific peptides of lung cancer and build a diagnostic model of MPE.

In comparison with other research exploring biomarkers of MPE by proteomics technology, our study has the following four advantages. First, our method—MALDI-TOF-MS combined with MB-WCX—was more suitable to the analysis of mixed biological samples and mainly focused on the low-molecular-weight and low-abundant proteins which include the peptides and protein hydrolysates associated with disease. Second, the MPE samples in training set were all definitely diagnosed by cytological smear, and thus the results were not influenced by paramalignant pleural effusion caused by airway obstruction of lung collapse, lymphatic obstruction, and systemic effects of cancer treatment [21]. Third, cytological results of all the selected MPE in training set showed adenocarcinoma cells. We once failed to build the model by comparing TPE samples with MPE samples that are mixed with different pathological types (adenocarcinoma, squamous cell carcinoma, and small cell lung cancer) because of the low recognition capability and cross-validation rate. We speculated that tumors with different pathological types have different biological behaviors, which is not conducive to the biomarker screening of a specific disease. Fourth, the benign PE were also strictly limited to inflammatory exudative PE samples, so we chose TPE for its high morbidity and difficulty to differentiate with MPE caused by lung cancer.

As a result, we found 28 different peptides (*P* < 0.05) in MPE and TPE samples by MALDI-TOF-MS. A total of 15 peptide peaks presented a higher peak area in MPE samples and can be the potential biomarkers in MPE of lung cancer. In this study, we successfully established a classification model by five peptides (917.37 Da, 4469.39 Da, 1466.5 Da, 4585.21 Da, and 3216.87 Da); the sensitivity and specificity of our MALDI-TOF-MS classification were 93.75% and 100% after the validation. All of the peptides were significantly different except the peptide 3216.87, because the panel of the peptides selected by ClinProTools software was an optimal combination cooperated with each other rather than the most important. Furthermore, the peptide 4469.39 was very close to the peptide 4,468.38 in our previous study which compared the different peptide profiles of serum between NSCLC patients and healthy people [18]; we speculated this peptide may be a secretory protein responsive to lung adenocarcinoma. It is also worth noticing that, in validation set, a patient was diagnosed with small cell lung cancer by pretreatment tumor-biopsy from pulmonary lesion, but his cytological result of MPE sample showed adenocarcinoma cell after systemic therapy, which probably resulted from intratumor heterogeneity or pathological transformation. The special MPE sample was classified as “malignant” by MALDI-TOF-MS classification, which indicated the classification model can recognize the MPE caused by pleural metastasis of lung adenocarcinoma correctly.

In this study, the detection rate of cytological smear was 69.70% (46/66), which was consistent with the results other previous studies showed [22, 23], while the detection rate of MALDI-TOF-MS classification model was 93.94% (31/33), which was statistically higher than traditional cytological method (*P* = 0.006). In addition, the cytology turnaround time was 3–5 days and required adequate sample volume as well as experienced pathologists, while, in contrast, the MALDI-TOF-MS method can be easily completed within a few hours and required less than 1 mL PE samples.

Despite no statistical difference between the specificity of MALDI-TOF-MS classification model and CEA, the sensitivity of MALDI-TOF-MS classification was significantly higher than CEA (*P* = 0.035). This suggested MALDI-TOF-MS classification was a superior method in diagnosis of MPE compared to traditional markers and we expected a better result by expanding the sample size because our model was a combination of five peptides rather than a single one.

Our present work explores a highly sensitive and specific MPE biomarker using the MALDI-TOF-MS technology combined with MB-WCX. These biomarkers provide a potential diagnostic platform for MPE of adenocarcinoma. Further studies with extended scale and other kinds of PE, such as PE of squamous cell lung cancer, small cell lung cancer, and breast cancer or pneumonia, is going to be conducted to explore new biomarkers of PE. In addition, the 5 peptide peaks differentiating MPE from TPE deserve to be further identified.

## 5. Conclusions

There were peptide differences between the MPE samples of lung cancer and TPE samples, and the different peptides may be the potential biomarkers of lung cancer. The results suggest MALDI-TOF-MS classification model which consists of five peptides (917.37 Da, 4469.39 Da, 1466.5 Da, 4585.21 Da, and 3216.87 Da) can predict MPE precisely and rapidly. Our MALDI-TOF-MS classification model of MPE has the potential for clinical application due to its accuracy and convenience.

## Figures and Tables

**Table 1:** Clinical and laboratorial characteristics of the patients with malignant and tuberculosis pleural effusion.

	Malignant pleural effusion *N* = 66	Tuberculosis pleural effusion *n* = 32	*P* value
Gender *n* (%)			0.275
Male	40 (60.60)	23 (71.88)	
Female	26 (39.40)	9 (28.12)	
Age (years)			<0.0001
Median (range)	61 (36–82)	29 (15–96)	
Smoking status *n* (%)			0.187
Ever-smoker	32 (48.48)	11 (34.38)	
Never-smoker	34 (51.52)	21 (65.62)	
Character *n* (%)			<0.0001
Bloody	42 (63.64)	2 (6.25)	
Nonbloody	24 (36.36)	30 (93.75)	
Cytopathology			ND
Positive	46 (69.70)	0 (0)	
Negative	20 (30.30)	32 (100)	
Protein level (g/L)	42.38 ± 9.09	44.97 ± 7.62	0.167
LDH level (U/L)	406.38 ± 328.59	394.88 ± 271.61	0.864
Cell count (×10^6^)	16801.00 ± 56862.44	10230.06 ± 13119.59	0.521

ND = not down.

**Table 2:** The 28 significant peptide peaks of malignant and tuberculosis pleural effusion in training set.

*m*/*z*	Peaks area of MPE ($\overset{-}{x}\pm S$)	Peaks area of TPE ($\overset{-}{x}\pm S$)	*P* value	State
917.37	22.25 ± 8.730	10.56 ± 4.680	<0.001	↑
4469.39	562.6 ± 326.2	184.1 ± 247.9	<0.001	↑
1466.5	23.23 ± 16.64	8.200 ± 4.920	<0.001	↑
2790.36	18.63 ± 11.20	9.450 ± 3.810	0.002	↑
861.51	42.70 ± 25.67	21.08 ± 13.80	0.003	↑
867.58	21.04 ± 19.09	6.640 ± 3.120	0.003	↑
3443.55	97.05 ± 118.3	683.1 ± 676.5	0.004	↓
805.31	10.11 ± 6.750	4.980 ± 2.890	0.004	↑
871.45	40.18 ± 21.32	21.96 ± 17.91	0.011	↑
3372.4	78.46 ± 73.91	468.1 ± 530.7	0.013	↓
3487.48	40.72 ± 55.10	307.2 ± 365.8	0.013	↓
4791.91	93.21 ± 128.6	13.31 ± 11.17	0.013	↑
4778.41	25.34 ± 31.98	6.270 ± 4.790	0.016	↑
3428.58	10.43 ± 7.260	54.79 ± 65.53	0.021	↓
4309.66	8.720 ± 3.500	13.89 ± 7.370	0.021	↓
3401.28	20.83 ± 16.57	102.1 ± 122.5	0.021	↓
3356.85	9.340 ± 4.420	39.34 ± 46.30	0.022	↓
1795.93	34.24 ± 27.78	17.47 ± 12.82	0.022	↑
4204.24	13.23 ± 11.91	27.21 ± 19.94	0.022	↓
3329.54	14.51 ± 6.160	44.98 ± 47.45	0.022	↓
877.63	87.30 ± 64.51	50.13 ± 27.82	0.025	↑
4215.49	10.39 ± 9.940	19.76 ± 13.65	0.030	↓
4585.21	21.55 ± 10.81	14.84 ± 7.360	0.032	↑
2234.19	48.70 ± 57.90	17.99 ± 22.07	0.035	↑
4247.85	88.60 ± 79.24	254.6 ± 282.2	0.036	↓
4356.04	52.36 ± 41.31	179.4 ± 226.5	0.044	↓
4540.29	32.01 ± 20.10	20.98 ± 11.72	0.044	↑
4327.25	5.980 ± 2.120	8.310 ± 3.930	0.044	↓

↑ signals showed a higher peak area in MPE.

↓ signals showed a lower peak area in MPE.

**Table 3:** The results of three statistical algorithms in ClinProTools software of training set.

Model name	Algorithms	Cross-validation	Recognition capability
GA-3	GA	77.09%	93.75%
GA-5	GA	76.07%	96.35%
GA-7	GA	78.29%	95.83%
SNN	SNN	81.06%	98.44%
QC	QC	80.17%	93.75%

GA: genetic algorithm. GA-3: number of neighbors is 3; GA-5: number of neighbors is 5; GA-7: number of neighbors is 7.

SNN: supervised neural network;

QC: quick classifier algorithm.

**Table 4:** The five peptides used to establish the diagnosis classification of MPE in ClinProTools software.

Index	Mass (Da)	Start mass (Da)	End mass (Da)	Weight
10	917.37	915.96	921.91	1.179690444559570
71	4469.39	4460.05	4479.55	0.924007763400121
13	1466.5	1462.24	1470.73	0.8662880291156875
73	4585.21	4566.07	4602.78	0.5132649678295391
45	3216.87	3207.9	3223.24	0.1573001919923568

**Table 5:** Blind test results of the model in validation set.

Confirmed samples	MALDI-TOF-MS classification			Total number	Sensitivity (%)	Specificity (%)	Accuracy (%)
	Labeled “malignant”	Labeled “benign”	Labeled “unclassifiable”				
MPE	15	0	1	16	93.75%	100.00%	96.15%
TPE	0	10	0	10			

**Table 6:** The comparison of detection rate between MALDI-TOF-MS classification and cytological smear method in pleural effusion.

Method	Number (%)			
	Result	Positive	Negative	Total number
MALDI-TOF-MS classification		31 (93.94)	2 (6.06)	33
Cytological smear method		46 (69.70)	20 (30.30)	66

*P* = 0.006 (3 patients with null spectra were excluded).

**Table 7:** The comparison of sensitivity of MALDI-TOF-MS classification and CEA detection in malignant pleural effusion.

Method	Number (%)			
	Result	Positive	Negative	Total number
MALDI-TOF-MS classification		15 (100.00)	0 (0.00)	15
CEA detection		21 (67.74)	10 (32.26)	31

*P* = 0.035 (1 patient with null spectra was excluded).

**Table 8:** The comparison of specificity of MALDI-TOF-MS classification and CEA detection in tuberculosis pleural effusion.

Method	Number			
	Result	Positive	Negative	Total number
MALDI-TOF-MS classification		0 (0.00)	10 (100.00)	10
CEA detection		9 (28.13)	23 (71.87)	32

*P* = 0.147.
